# Bisdemethoxycurcumin Enhances the Sensitivity of Non-small Cell Lung Cancer Cells to Icotinib via Dual Induction of Autophagy and Apoptosis

**DOI:** 10.7150/ijbs.40042

**Published:** 2020-03-05

**Authors:** Min Xiang, He-Guo Jiang, Yang Shu, Yu-Jiao Chen, Jun Jin, Yu-Min Zhu, Mei-Yu Li, Jian-Nong Wu, Jian Li

**Affiliations:** 1 Department of Clinical Laboratory, Affiliated Hospital of Jiangsu University, Zhenjiang 212001, China.; 2 Department of Pulmonary Medicine, Affiliated Hospital of Jiangsu University, Zhenjiang 212001, China.; 3 Center of Medical Experiment, Affiliated Hospital of Jiangsu University, Zhenjiang, 212001, China.; 4 Department of pathology, Affiliated Hospital of Jiangsu University, Zhenjiang 212001, China.

**Keywords:** non-small cell lung cancer cells, icotinib, resistance, bisdemethoxycurcumin (BDMC), apoptosis, autophagy

## Abstract

Non-small cell lung cancer (NSCLC) with epidermal growth factor receptor (EGFR) wild-type is intrinsic resistance to EGFR-tyrosine kinase inhibitors (TKIs). In this study, we assessed whether the combination of bisdemethoxycurcumin (BDMC) and icotinib could surmount primary EGFR-TKI resistance in NSCLC cells and investigated its molecular mechanism. Results showed that the combination of BDMC and icotinib produced potently synergistic growth inhibitory effect on primary EGFR-TKI-resistant NSCLC cell lines H460 (EGFR wild-type and K-ras mutation) and H1781 (EGFR wild-type and Her2 mutation). Compared with BDMC or icotinib alone, the two drug combination induced more significant apoptosis and autophagy via suppressing EGFR activity and interaction of Sp1 and HDCA1/HDCA2, which was accompanied by accumulation of reactive oxygen species (ROS), induction of DNA damage, and inhibition of cell migration and invasion. ROS inhibitor (NAC) and autophagy inhibitors (CQ or 3-MA) partially reversed BDMC plus icotinib-induced growth inhibitory effect on the NSCLC cells. Meanwhile, co-treatment with NAC attenuated the two drug combination-induced autophagy, apoptosis, DNA damage and decrease of cell migration and invasion ability. Also, 3-MA or CQ can abate the combination treatment-induced apoptosis and DNA damage, suggesting that there is crosstalk between different signaling pathways in the effect produced by the combination treatment. Our data indicate that BMDC has the potential to improve the treatment of primary EGFR-TKI resistant NISCLC that cannot be controlled with single-target agent, such as icotinib.

## Introduction

Lung cancer remains the top cause of cancer death worldwide and non-small cell lung cancer (NSCLC) accounts for > 80% of the recorded cases 1. With in-depth studies of cancer related signaling pathways, epidermal growth factor receptor (EGFR)-dependent pathway was revealed to play important roles in the development and progression in NSCLC 2. EGFR-tyrosine kinase inhibitors (TKIs) such as gefitinib and erlotinib have been demonstrated to increase response rates, extend survival and improve quality of life as compared with standard platinum-based chemotherapy in patients with advanced NSCLC with activating EGFR mutation 3,4. Icotinib which is another first-generation EGFR-TKI, has been approved as the first-line therapy in NSCLC patients with activating EGFR mutation by China Food and Drug Administration (CDFA) 5. Compared with the gefitinib, icotinib shows similar clinical therapeutic efficacy and better safety 6. However, almost all patients with initial sensitivity to EGFR-TKI treatment inevitably developed acquired drug resistance that results in therapeutic failure 7. In addition, patients with EGFR wild-type NSCLC are intrinsic resistance to EGFR-TIKs 8, especially for concurrent with KRAS mutation or HER2 mutation 9, 10. How to target to the EGFR-TKI insensitive EGFR wild-type NSCLC is largely undetermined. Hence, there is an urgent demand to develop new combination of treatment to simultaneously target multiple pathways, and thereby enhancing the efficacy of EGFR-TKI in treating NSCLC, in particular, the EGFR wild-type NSCLC.

It is well known that curcumin is able to suppress cancer cell proliferation, invasion, angiogenesis, and metastasis through a diversity of signaling pathways, involving induction of apoptosis and autophagy 11. However, relative poor stability of curcumin has been highlighted as one of the major problem in therapeutic applications 12. To enhance metabolic stability and antiproliferation activity against human cancer cells, various curcumin analogs have been synthesized. Besidemethoxycurcumin (BDMC), a demethoxy derivative of curcumin, exhibits significantly different antimutagenic, anticancer and antimetastasis activities, although it differs from curcumin in the chemical structure only with regard to methoxy substitution 11. Importantly, BDMC has been showed to be more stable than curcumin in physiological medium 12. EGFR-TKIs have been reported to induce autophagy in NSCLC cells 13. We recently found that survivin inhibitor YM155 sensitize NSCLC cells to EGFR-TKIs through autophagy induction mediated by downregulating survivin expression and the AKT/mTOR pathway 14. We hypothesized that BDMC reverse intrinsic icotinib resistance in NSCLC cells with EGFR wild-type and K-ras mutation or Her 2 mutation through induction of autophagy-related cell death and apoptosis. In this study, we examined the effect of BDMC plus icotinib on primary TKI resistant NSCLC cells, and investigated the underlying molecular mechanism of icotinib sensitization by BDMC in NSCLC cells expressing EGFR wild-type and K-ras mutation or Her2 mutation.

## Materials and methods

### Cell cultures and reagents

Three EGFR-TKI resistant NSCLC cell lines A549 (EGFR wild-type and KRAS mutation), H460 (EGFR wild-type and KRAS mutation), H1781 (HER2 G776 INSv-G/C mutation), and an EGFR-TKI sensitive NSCLC cell line HCC4006 (EGFR exon 19 deletion) were obtained from American Type Culture Collection (ATCC, Manassas, VA). The cells were cultured in 10% fetal bovine serum-supplemented RPMI 1640 medium (Sigma-Aldrich, St Louis, MO) and 1% penicilin-streptomycin under a humidified atmosphere of 5% CO_2_ at 37℃. All cell lines were routinely tested and were negative of mycoplasma contamination.

For agents and antibodies used in this study see Supplementary materials and methods.

### Cell survival and viability assays

Cell survival was determined using methylene blue stain, as our previously reported 15. Cell viability was determined using the cell counting-kit (CCK-8, DOJNDO, Japan) assay according to manufacturer`s instructions, as previously described 15. For more details see Supplementary materials and methods. Growth inhibition was expressed as the percentage of surviving cells (which was considered as 100% viability in control). The IC_50_ value was the concentration resulting in 50% cell growth inhibition by a 48-h exposure to drugs compared with untreated control cells. Interactions between icotinib and BDMC were expressed as the combination index (CI) by the CompuSyn software: (< 1 represents synergistic effects; 1 represents additive effect; and > 1 represents antagonistic affect 16.

### Clonogenic assay and cell apoptosis analysis

Cell colony formation ability was measured by clonogenic assay as previously reported 17. Cell apoptosis was determined using flow cytometry with the Annexin V-FITC PI staining in accordance with the manufacturer's instructions as previously described 17. Cell sub-G1 population was analyzed by flow cytometry as previously reported 18. For more details see Supplementary materials and methods.

### Immunoblot assay and real-time quantitative PCR analysis

Before and after treatment with indicated drugs, or transfections with the siRNAs against indicated genes, whole lysate proteins extracted from the cells were prepared and analyzed by standard immunoblot assay as previously reported 17,18.

For real-time quantitative PCR analysis, total RNA was extracted from cell samples using Trizol reagents (Invitrogen) following the manufacturers' instructions. RNA was reverse transcribed into cDNA using qScript (Quanta Biosciences) following the manufacturer's instructions and the reactions were run on an ABI 7500 Fast real-time PCR systems, as previously reported 17. For more details see Supplementary materials and methods.

### siRNA transfection

All siRNA reagents used in this study were purchased from Guangzhou Riboio Co., Ltd (China). Cells were seeded at for a 6-well plate and cultured for 24 h. Then the cells were transfected with Lipofectamine 2000 reagents (Invitrogen) according to the manufacturer's instructions, as previously described 17. The efficacy of transfection (including siSp1, siSp3, siHDAC1, siHDAC2, siVDAC1 and siBeclin-1) were verified by immunoblot assay (Figure S1D-S1F).

### Plasmid transient transfection

The Sp1 and control plasmid DNA (PCMV6 XL8) used in this study were purchased from OriGene (Rockville, MD). For transient transfection, 5 × 10^5^ cells per well were seeded into 6-wells plates and transiently transfection with 2 μg of Sp1 plasmids using Lipofectamine 2000 transfection reagent (Invitrogen Calsbad, CA) according to the manufacturer's instructions. Briefly, plasmid DNA (2 μg) was diluted into 100 μL of RPMI 1640 media. The two solutions were then mixed together and incubated at room temperature for 30 min. The total mixed was added to each well (6-well plate) containing 800 μL RPMI 1640 media. Following transfection, cells were allowed to recover for 24 h, and then state the treatment.

### Luciferase activity assay

Cells were transfectcd with an HDCA1 promotor luciferase reporter plasmid using Lipofectamine 2000 transfection reagent (Invitrogen), following the manufacturers' protocol. After transfection, cells were cultured in 24-well plates in 10% FBS-supplemented RBMI 1640 for 48 h, and subject to the indicated drugs for 48 h, collected and lysed with passive lysis buffer (Promega, Madison, WI). Aliquosts of the lysates (50 μL) were added to media, and luciferase activity was monitored after adding 100 μL of luciferase substrate (Promega) each well by using a MicroLumatPlus LB96V Luminometer (Bethold Technologies) with the WinGlow Software package.

### Measurement of ROS

Intracellular reactive oxygen species (ROS) were measured with the cell permeant prob carboxy-H_2_DCFDA (5-(and-6)-carboxy-2'7'-dichlorodihydrofluorescein diacetate) from Invitrogen (Calsbad, CA). Following treatment with various drugs at doses as indicated for 48 h, cells seeded on 6-well plates were loaded with 10 mM of carboxy-H_2_DCFDA for 1 h, washed once with serum-free medium, and analyzed for ROS levels by BD Accuri C6 Flow Cytometer using the FL1 channel. Analysis of data was performed by BD Accuri CFlow Software (ACFS) (set at 480 nm and 525 nm extation and emission wavelengths, respectively).

### Immunofluorescent staining and comet assay

LC3-II puncta was detected by immunofluorescent staining, as previously described 14,17. The intracellular localization of the proteins, such as γ-H2AX, p-ATM and 53-BP1, were determined by immuofluorescent analysis as described previously 14,17,18. The formation of acidic vesicular organelles (AVOs) was detected using monodansylcadaverine (MDC) staining. For more details see Supplementary materials and methods.

The modified alkaline comet assay was performed to determine DNA damage following the manufacturers' protocol, as previously reported 15.

### Cell migration and invasion assays

Cell migration was determined by wound healing assay. Cell invasion was examined by membrane transwell culture system. For more details see Supplementary materials and methods.

### *In vivo* NSCLC cell xenograft experiment

BALBL/c nude mice (specific pathogen free grade, male, 5-6 weeks of age) were obtained from the Model Animal Research Center of Nanjing University (Nanjing, China). All animal procedures were performed in accordance with the protocols approved by the Institutional Animal Care and Use Committee of the Jiangsu University. H460 cells (6x10^6^ cells per mouse) were subcutaneously injected into the right flanks. When tumor reached more than 5 mm in diameter, mice were randomly allocated into 4 groups (5 mice/per group) to receive either vehicle control, icotinib (125 mg/kg, one daily) alone, BDMC (100 mg/kg, once daily) alone, or icotinib plus BDMC. Icotinib was suspended in saline and BDMC was prepared properly in propylene glycol and administered orally by gavage. Tumor volume was measured every 4 days using calipers, and calculated according to the equation (lenth **×** width^2^)/2. After 21 days, the mice were killed and the tumors were excised and weighted, and stored in -80℃ until further analysis.

### Statistical analysis

All the experiments were performed in triplicate, and the results are expressed as mean ± SEM. For comparisons of two groups, a two-tailed unpaired *t*-test was used; for comparisons of multiple groups, one-way ANOVA analysis followed by Tukey post hoc tests was applied. Statistical significance was set as *P* < 0.05.

## Results

### BDMC synergistically enhances antitumor activity of icotinib in NSCLC cells

**Figure 1:**
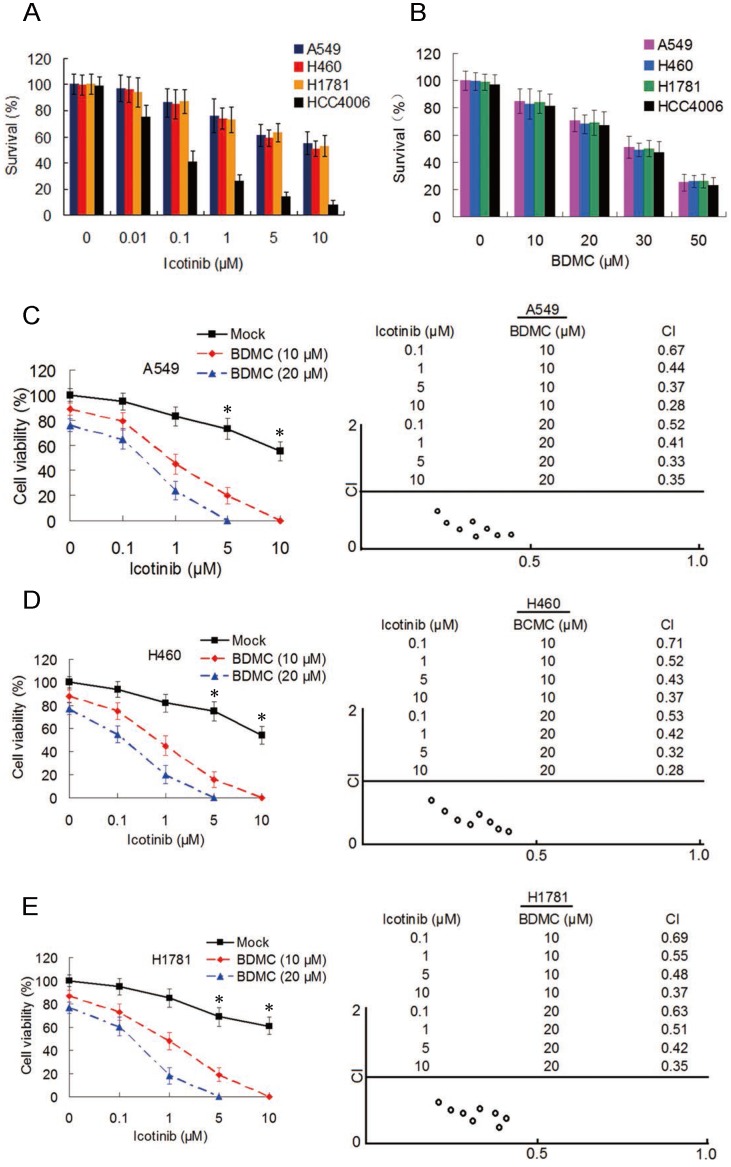
** BDMC potentiates antitumor activity of icotinib in EGFR-TKI-resistant NSCLC cells.** (**A**) A549, H460, H1781 and HCC4006 cell lines were treated with icotinib at indicated doses , or (**B**) with BDMC at indicated doses for 48 h. Cell survival was detected by the methylene blue staining as described in Materials and Methods. (**C-E**) A549, H460 and H1781 cells were treated with icotinib and/or BDMC at indicated doses for 48 h. Cell viability wad determined by CCK-8 assay. The results are expressed as the percentage of survival cells in drug-treated cells relative to DMSO-treated control cells. *P < 0.05 vs BDMC 10 µM and 20 µM. CI values for the combination of icotinib and BDMC were calculated using the Calcusyn software (Cambrige, UK) as described in Materials and Methods.

To examine the effect of icotinib plus BDMC in EGFR-TRI-resistant NSCLC cells with different EGFR characteristics, three primary TKI-resistant cell lines, including A549, H460 and H1781 were used, which showed an overall pattern of increased resistance when incubated with icotinib for 48 h (IC_50_ > 20 μM for the three cell lines), compared with TKI-sensitive cell line HCC4006 (IC_50_ = 55.4 nM) (Figure 1A). As expect, BDMC inhibits cell survival with no difference in TKI-resistant and -sensitive cell lines in a dose-dependent manner (Figure 1B). Then the TKI-resistant cells were treated with increasing concentrations of icotinib and/or BDMC for 48 h, and cell viability was measured by CCK-8 assay. Compared with icotinib alone, all cells treated with icotinib plus BDMC exhibited markedly decreased viability (Figure 1C-1E). Almost all of the CI values at various combination were less than 0.7, indicating a strong synergy of BDMC and icotinib in these primary TKI-resistant NSCLC cell. To examine whether BDMC and icotinib also hold synergistic effects in TKI-sensitive cells, we tested HCC4006 cells with various doses of icotinib and BDMC. Although icotinib plus BDMC reduced cell viability compared with icotinib alone, most of the CI values of this drug pair at different combination in HCC4006 cells were close to 1 (Figure S1A and S1B), suggesting that sensitization degree by icotinib plus BDMC in HCC4006 cells was inferior to TKI-resistant cell lines. In addition, we observed significant reductions in the colony formation capacities of these NSCLC cells treated with icotinib plus BDMC compared to either of the two drugs (Figure S1C). Collectively, the results indicate that BDMC can potentiate growth inhibitory effect of icotinib on TKI-resistant NSCLC cells.

Because A549 and H460 cells lines have same EGFR characteristics, we used H460 and H1781 cell lines in the subsequent experiment.

### Co-treatment with icotinib and BDMC suppresses EGFR activity by downregulating Sp transcription factors and HDACs

**Figure 2:**
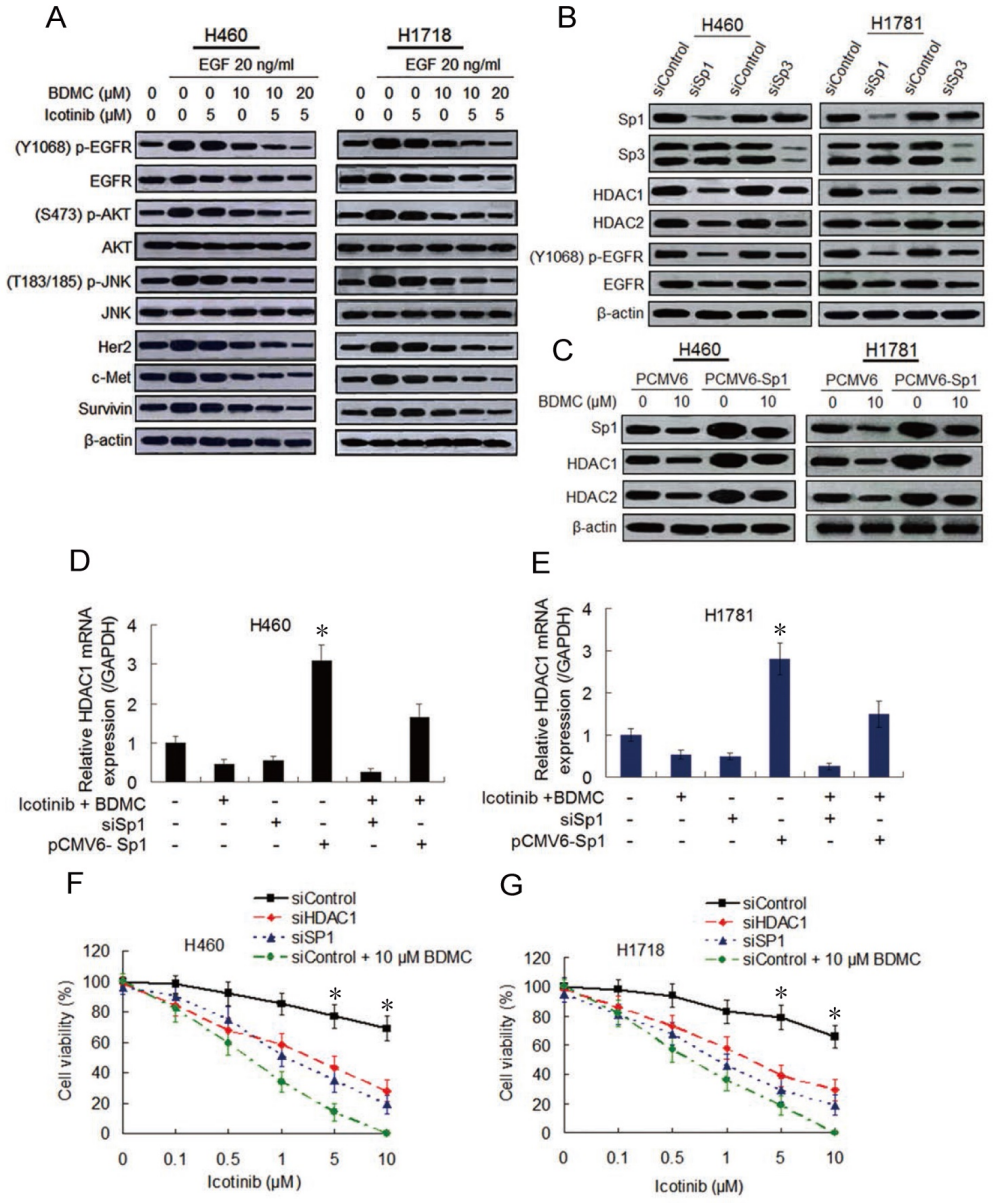
** Combination of icotinib and BDMC suppresses EGFR activity by downregulating expressions of SP1 and HDAC1/HDAC2.** (**A**) H460 and H1781 cells were pre-treated with icotinib and/or BDMC at the doses indicated for 6 h, and then stimulated with EGF at indicated dose for 1 h. Then cell lysates were prepared and subjected to immunoblot assay to detect the protein expressions as indicated. (**B**) Expressions of Sp1/Sp3 and other indicated proteins in control and SP1or SP3 knocked down cells were detected by immunoblot assay in H460 and H1781 cells. (**C**) H460 and H1781 cells were transfected with Sp1 plasmids (PCMV6-Sp1) and control plasmids DNA (PCMV6). After 8 h, cells were treated with BDMC at indicated doses for an additional 48 h. Then the expressions of Sp1 and HDAC1/HDAC2 proteins were detected by immunoblot assay. (**D** and **E**) Before and after transfection with or without siSp1 or PCMV6-Sp1, H460 and H1781 cells were treated with icotinib (5 µM) plus BDMC (10 µM) for 48 h, and HDCA1 mRNA expression was analyzed by real-time quantitative-PCR, as described in Materials and Methods. *P < 0.05 vs other every treatment. (**F** and **G**) Following transfection with siControl or siSp1 or siHDAC1, H460 and H1781 cells were treated with icotinib at indicated doses, or icotinib plus BDMC at indicated doses for 48 h, then CCK-8 assay was performed to determine cell viability. *P < 0.05 vs siSp1 and siHDAC1.

Then we examine whether BDMC can enhance the inhibitory effect of icotinib on EGFR activity. Immunoblot analysis show that the cells pre-treated with icotinib or BDMC alone for 6 h and then stimulated with EGF (20 ng/mL) for 1 h displayed slight reduction of EGFR phosphorylation levels, and the endogenous EGFR were also decreased to a small extent. However, icotinib plus BDMC significantly downregulated phosphorylated (p-) and endogenous EGFR levels stimulated by EGF in TKI-resistant H460 and H1718 cells, with reduction of p-EGFR/EGFR ratio (Figure 2A and S2A). Similar changes of p-EGFR and endogenous EGFR levels in TKI-sensitive HCC4006 cells were observed (Figure S2B and S2C). Since EGFR expression is regulated by multiple factors, including specific protein (Sp) transcioption factors through interaction with HDAC1 and HDCA2 in cancer cells 19, 20, we tested the effect of Sp1 and Sp3 knockdown on EGFR activity and HDCA expressions. We found that Sp1 knockdown clearly inhibited expressions of HDCA1, HDCA2 and p-EGFR and ratio of p-EGFR/EGFR, whereas Sp3 knockdown lightly downregulated expressions of these proteins (Figure 2B and S2D). Moreover, icotinib plus BDMC obviously suppressed HDCA1 and HDCA2 expressions and increased global histone acetylation levels, but HDCA3 and HDCA4 expressions were not affected by the combination treatment (Figure S3A). Meantime icotinib plus BDMC clearly inhibited Sp1-dependent protein expressions, such as Her2, c-Met and survivin, and EGFR downstream protein phosphorylations (Figure 2A and S2B).

To further demonstrate the effect of Sp1 and HDCA1/HDCA2 on EGFR activity after icotinib and BDMC treatment, Sp1 ectopic overexpressed H460 and H1781 cell lines were established by plasmid transfection. Sp1 overexpression increased HDCA1/HDCA2 expressions, abated inhibitory effect of BDMC on HDCA1/HDCA2 expressions (Figure 2C). Meanwhile, knockdown of HDCA1 noticeably downregulated levels of p-EGFR and endogenous EGFR in H460 and H1781 cells, whereas EGFR activity was moderately depressed by HDCA2 knockdown (Figure S3B). In addition, quantitative PCR analysis showed that Sp1 overexpression markedly elevated HDCA1 mRNA levels and retarded decline of HDCA1 mRNA level induced by BDMC plus icotinib (Figure 2D and 2E). And Sp1 knockdown resulted in a significant decrease of HDCA1 mRNA levels, which was similar to the results caused by icotinib plus BDMC treatment (Figure 2D and 2E). Likewise, luciferase assay demonstrated that HDCA1 promotor activity was markedly enhanced by Sp1 overexpression and suppressed by Sp1 knockdown (Figure S3C and S3D). Since HDCA1/HDCA2 promotors are regulated by Sp1 and Sp3 through binding to GC boxes of HDCA1/HDCA2 21, we treated the cells with mithramycin A (MIT), a reagent that blocks Sp1 and Sp3 binding to GC boxes. Quantitative PCR analysis showed that treatment with increasing doses of MIT decreased HDCA1/HDCA2 mRNA levels in the two cell lines, and the protein levels of HDCA1/HDCA2 were also reduced (Figure S3E and S3F). Furthermore, knockdown of Sp1 or HDCA1 sensitized the two TKI-resistant cell lines to icotinib, although the sensitization degree was inferior to BDMC (Figure 2F and 2G). Taken together, the data indicate that BDMC plus icotinib caused synergistic growth inhibition in the TKI-resistant NSCLC cells depend on EGFR activity downregulation mediated by suppressing Sp1 expression and the interaction of Sp1 and HDCA1/HDCA2.

### Combination treatment with icotinib and BDMC activates ROS generation and induces apoptosis by suppressing VDAC1

**Figure 3:**
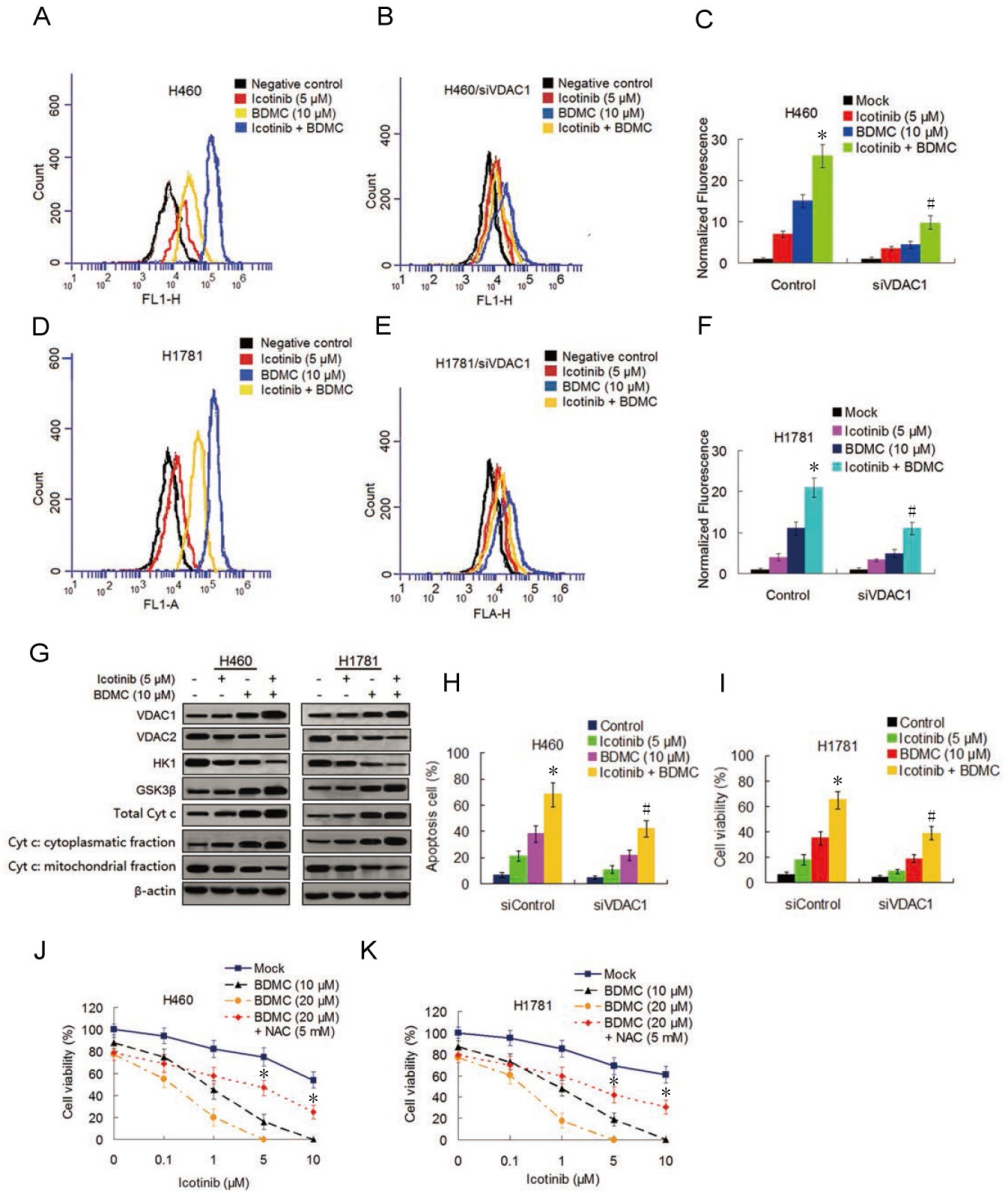
** Icotinib plus BDMC activates ROS generation and modulates VDAC and mitochondrial homestasis.** (**A** and** D**) Before and (**B** and **E**) after transfection with siVDAC1, H460 and H1781 cells were treated with icotinib and/or BDMC at indicated doses for 48 h and induction of ROS was measured by BD Accuri CFlow software (ACFS), as described in Materials and Methods. The intensity of ROS fluorescence in Mock treated control was defined as 1. (**C** and** F**) The quantative analysis of the intensity of ROS fluorescenceof. *P < 0.05 versus icotinib or BDMC; # *P* < 0.05 vs icotinib + BDMC in Control. (**G**) H460 and H1781 cells were treated with icotinib and/or BDMC at indicated doses for 48 h, and subjected to immunoblot assay to determine the protein expressions indicated. (**H** and **I**) After tranfection with siVDAC1 or siControl, H460 and H1781 cells were treated with icotinib and/or BDMC at indicated doses for 48 h, then flow cytometry was carried out to determine the percentage of apoptotic cells. *P < 0.05 vs icotinib or BDMC, # *P* < 0.05 versus icotinib + BDMC in Control. (**J** and** K)** H460 and H1781 cells were treated with icotinib alone, or icotinib plus BDMC at indicated doses, or the two combination plus NAC at indicated doses for 48 h, cell viability was determined by CCK-8 assay. *P < 0.05 vs BDMC 20 µM.

Curcumin was reported to induce reactive oxygen species (ROS) generation 22 we tested the ability of icotinib and BDMC to produce ROS in H460 and H1781 cells and its effect on the cell growth inhibition. As shown in Figure 3A-3F, icotinib plus BDMC dramatically induced ROS generation compared to icotinib or BDMC alone. When VDAC1, the mitochondrial porin voltage-dependent anion-selective channel protein 1 which is a key player in mitochontrial-mediated apoptosis and involved in ROS generation 23, was knocked down by siRNA transfection in H460 and H1781 cells, icotinib plus BDMC-induced ROS generation were remarkably decreased (Figure 3B, 3C and 3E, 3F). Immunoblot assay showed that icotinib plus BDMC treatment led to upregulation of VDCA1 expression (Figure 3G). Conversely, the expressions of the enzyme HK1 that interacts with VDAC1 to suppress its oligomerization and consequent ROS release from mitochondria was clearly reduced, and the expression of anti-apoptosis mitochondrial porin VDAC2 was also inhibited in these cells upon icotinib plus BDMC treatment (Figure 3G). Concurrently we observed increased expression of the Akt-negative regulated enzyme GSK-3β (Figure 3G) which can cause the oligomerization and activation of VDAC1 by inducing the release of HK1 from the mononer VDAC1 24. Additionally we found that compared with single drug treatment, icotinib plus BDMC resulted in upregulated expression of total cytochrome c, together with an increase in the cytochrome c cytoplasmic fraction paralleled by a decrease in the mitochondrial fraction on these cells (Figure 3G).

To further determine whether VDAC1 modulation is involved in the synergistic interaction between icotinib and BDMC, we evaluated the effect of VDAC1 knockdown on apoptosis. As shown in Figure 3H and I, knockdown of VDAC1 retarded increase of apoptosis cells induced by icotinib plus BDMC treatment. Moreover, the inhibitory effect of icotinib plus BDMC on cell viability was reversed partially by co-treatment with antioxidant N-acetylcysteine (NAC) (Figure 3J and 3K). Altogether, the results indicate that the synergistic effect of icotinib and BDMD on NSCLC cell growth inhibition can be partially attributed to upregulation of VDAC1, accumulation of ROS and mitochondrial-mediated apoptosis.

### Combination of icotinib and BDMC induces autophagic cell death and autophagy-dependent and -undependent apoptosis

**Figure 4:**
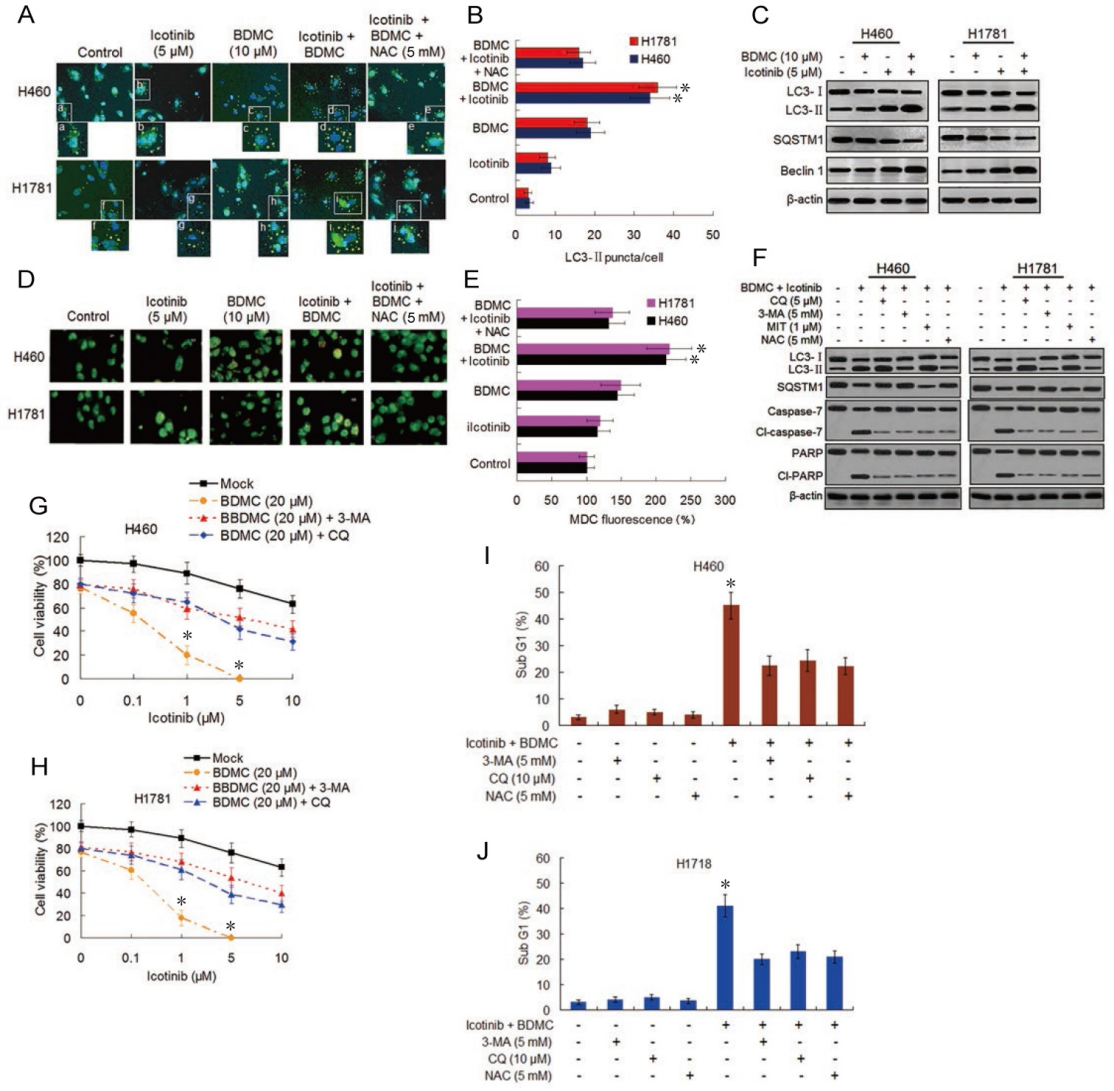
** Combination of icotinib and BDMC enhances induction of autophagy and apoptosis.** (**A** and** B)** H460 and H1781 cells were treated with icotinib and/or BDMC, or the two drugs combination plus NAC at indicated doses for 24 h, then the LC3-II puncta (yellow puncta as shown in the pictures) was detected by immunofluorescent staining and imaged using confocal microscope. The number of LC3-II puncta/cell was quantified by ImagePro plus 5.1 software. *P < 0.05 vs icotinib + BDMC + NAC or BDMC alone. (**C**) H460 and H1781 cells were treated with icotinib and/or BDMC at indicated doses for 48 h, and subjected to immunoblot assay to determine the protein expressions as indicated. (**D and E**) The two cell lines were treated with various drugs as described in (**A**) and (**B**), then AVOs (orange color puncta as shown in the pictures) in cells were detected by MDC staining and observed under a fluorescence microscope. The intensity of MDC fluorescence in untreated control was defined as 100%. *P < 0.05 vs icotinib + BDMC + NAC or BDMC alone. (**F**) The two cell lines were treated with icotinib (5 µM) plus BDMC (10 µM), or the two drugs combination plus CQ, or 3-MA, or MIT, or NAC at indicated doses for 48 h, and then subjected to immunoblot analysis to detect the protein expressions as indicated. (**G and H**) H460 and H1781 cells were treated with icotinib and/or BDMC at indicated doses, or the two drugs combination plus 3-MA (5 mM), or CQ (5 µM) for 48 h, then cell viability were determined by CCK-8 assay. *P < 0.05 vs 20 µM BDMC. (**I and J**) The two cell lines were treated with icotinib (5 µM) plus BDMC (10 µM), or the two combination plus 3-MA, CQ or plus NAC at indicated doses for 48 h, then sub G1 cell population were measured by flow cytometry. *P < 0.05 vs icotinib + BDMC in combination with 3-MA, or CQ, or NAC.

Both EGFR-TKI and curcumin have been demonstrated to induce autophagy 15,18. Oxidative stress was shown to be one triggering force for the activation of autophagy in cancer cells 25. Thus we evaluated the effect of icotinib plus BDMC treatment on autophagy induction. As shown in Figure 4A and 4B, compared with control cells, the slight increase of LC3-Ⅱpuncta formation by icotinib alone was found in H460 and H1781 cells, was also observed with BDMC alone, and was further increased by combination treatment with the two drugs, indicating increased formation of autophagosome. Icotinib plus BDMC also augmented the formation of fluorescent puncta in the MDC-stained cells, as indicated by increased fluorescence intensity values (Figure 4D and 4E), which are indicative of acidic vesicular organelles (AVOs) including lysosome and autolysosome. The increased formation of LC3-Ⅱ puncta and AVOs induced by icotinib plus BDMC can be blocked by addition of NAC, suggesting that the combination treatment-induced autophagy is at least in part ROS dependent. Meanwhile, icotinib plus BDMC increased the conversion of LC3-Ⅱ and expression of Becline-1 and decreased SQSTM1 levels (Figure 4C), which are molecular markers for autophagy. Moreover, increased LC3-Ⅱ accumulation and decreased SQSTM1 expression caused by icotinib plus BDMC were attenuated by 3-MA and NAC, and augmented by CQ and MIT (Figure 4F), signifying that the combination treatment produced ROS accumulation and EGFR activity reduction mediated by inhibiting SP1 and HDCA1/HDCA2 interaction are involved in autophagy induction. Furthermore, the combination treatment caused growth inhibitory effect in the cells can be reversed partially by CQ and 3-MA (Figure 4G and 4H), indicating that autophagic cell death is a mechanism of synergism of BDMC to icotinib. In addition, icotinib and BDMC combination-induced increases of cleaved-caspase 7 and -PARP as well as cell sub G1 cell population were abated by 3-MA, CQ, and NAC respectively (Figure 4F, 4I-4J), also increased sub G1 cell population were retarded by knockdown of beclin-1 (S4A and S4B), suggesting that the combination treatment-induced apoptosis depend partially on autophagy activation and oxidative stress. Additionally, repression of colony formation in the cells treated with icotinib plus BDMC can be reversed in part by 3-MA or NAC (Figure S4C and S4D), further indicating that autophagy and oxidative stress are involved in synergistic lethal effect of the two drug combination on the TKI-resistant NSCLC cells.

### Icotinib and BDMC combination augments DNA damage that depend on autophagy induction and ROS generation

**Figure 5:**
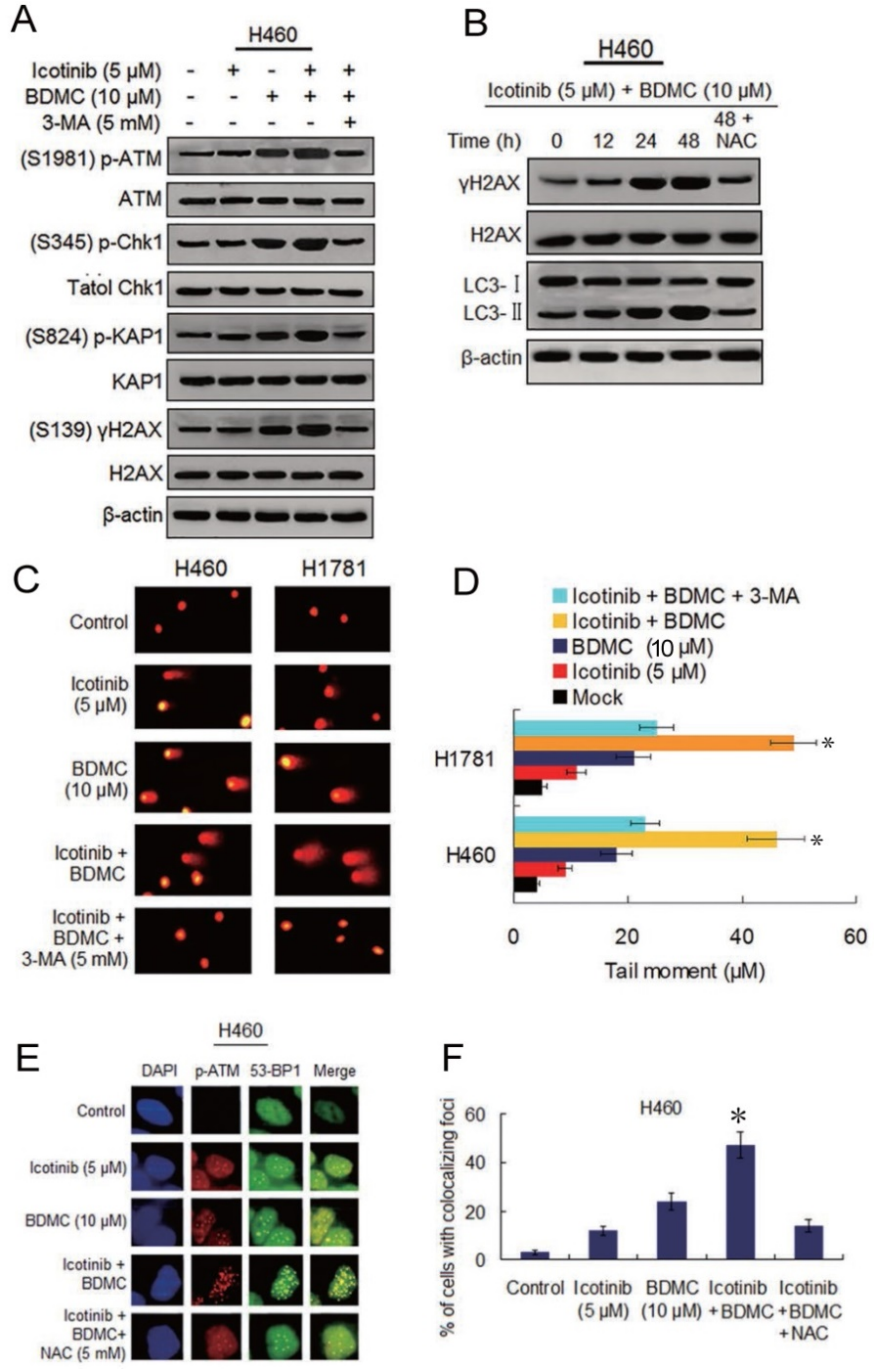
** Combination of icotinib and BDMC induces marked DNA damage that depend on autophagy and oxidative stress.** (**A**) H460 cells were treated with icotinib and/or BDMC, or the two combinations plus 3-MA at indicated doses for 48h, then subjected to immunoblot assay to determine phosphorylated (p) or total protein expressions as indicated. (**B**) H460 cells were treated with icotinib plus BDMC at indicated doses for 12, 24, and 48 h, or icotinib plus BDMC in combination with NAC at indicated doses for 48 h, and then subjected to immunoblot assay to detect the proteins expressions as indicated. (**C** and** D**) H460 and H1781 cells were treated with icotinib and/or BDMC, or the two drug combination plus 3-MA at indicated doses for 48 h. Then comet assay was performed to detect DNA double strand breaks, and images show detectable comet tail when visualized under a fluorescent microscopre. Tail moment in the cells were quantified using Comet Score software version 1.5. *P < 0.05 vs icotinib + BDMC + 3-MA, or BDMC. (**E** and** F**) H460 cells were treated with icotinib or BDMC alone, or icotinib plus BDMC, or the two combination plus NAC at indicated doses for 12 h, then colocalizations of p-ATM and 53-BP1 foci were determined by immunofluorescent staining using specific antibodies. Frequency of positive colocalization (the percentages of cells exhibiting more than 10 foci in the nucleus on confocal images) was analyzed by SlideBook 5.5 software. (*P < 0.01 versus icotinib + BDMC + NAC, or BDMC).

Seeing that BDMC can cause DNA damage and depress DNA repair capability 17, we investigated the ability of the two drug combination to induce DNA damage and its relationship with autophagy and oxidative stress. Since the ataxia-telangiectasia mutant (ATM) is known to be a major player of the DNA damage response (DDR) 26, we tested icotinib and BDMC combination induced phosphorylation levels of the ATM and its downstream proteins. As shown in Figure 5A, the slight or moderate upregulated phosphorylation of ATM, Chk1, KAP1 and γ-H2AX were observed in H460 cells following icotinib or BDMC treatment alone, the two combination treatment markedly elevated the phosphorylation levels of these proteins, which were attenuated by adding 3-MA. Likewise, icotinib plus BDMC resulted in an obvious increase of γ-H2AX foci positive cells, which was also reduced by addition of 3-MA (Figure S5A and S5C). Moreover, a time dependent increase in γ-H2AX expression was seen in H460 cells after treatment with icotinib plus BDMC, in paralleled by increase of LC3-Ⅱ conversion, which were attenuated by NAC (Figure 5B).

The similar results were obtained in H1781 cells (Figure S5B-S5E). Consistent with the findings, comet assay showed that icotinib plus BDMC treated H460 and H1781 cells exhibited notably prolonged tail moment in comparison with the cells treated with icotinib or BDMC alone (Figure 5C and 5D). Because incellular localization of phosphorylated ATM and 53-BP1 proteins are well characterized markers of DNA double-strand breaks (DSBs) 27, we tested the foci marked by p-ATM colocalized with 53-BP1 in H460 cells. The results showed that the percentage of H460 cells exhibiting p-ATM and 53-BP1-colocalized foci reached a higher levels 24 h after treatment with icotinib plus BDMC (Fig. 5E and F), implying that DSB damage in these cells was induced to a large degree, compared to treatment with icotinib or BDMC alone. Collectively, these results indicate that icotinib and BDMC synergistically induce DNA damage in the TKI-resistant NSCLC cells, which is dependent on autophagy induction and ROS accumulation, thereby promoting cell death.

### The two drug combination effectively inhibit cell migration and invasion by suppressing MMPs and VEGF and upregulating HLJ1 and E-cadherin

**Figure 6:**
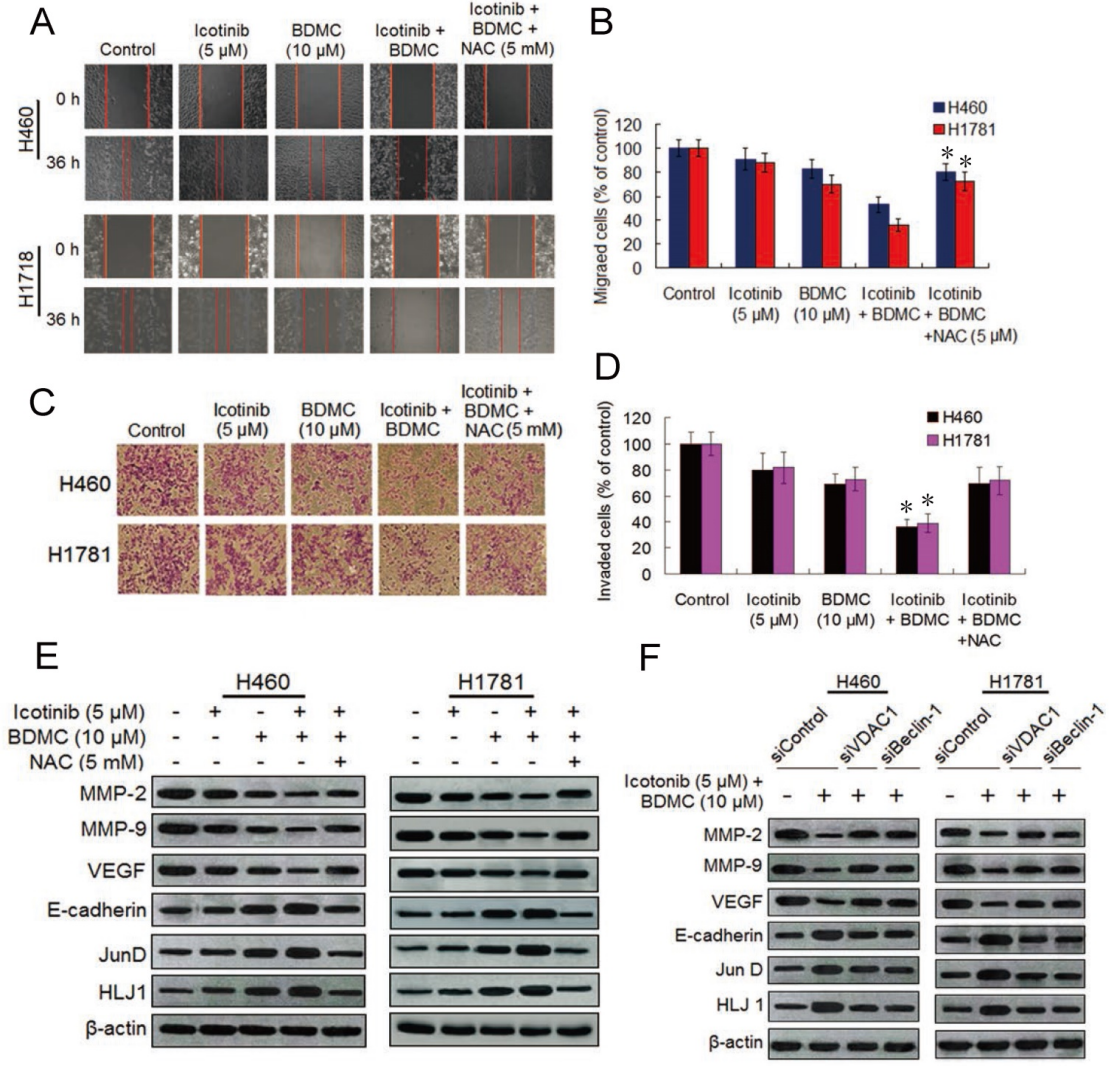
** Icotinib plus BDMC inhibits cell migration and invasion in EGFR-TKI resistant NSCLC cells**. (**A** and** B**) The effect of icotinib plus BDMC on cell migration was measured using the wound-healing assay. Confluent monolayers of H460 and H1781 cells were scratched, and then treated with icotinib and/or BDMC, or the two combination plus NAC at indicated doses for 36 h. Images were acquired immediately (0 h) and at 36 h after wounding. Cell migrating into the wound area were counted based on the dash line as time zero. The quantity of cell migration to the wound area was defined as 100% in control at 36 h time points. *P < 0.05 versus icotinib + BDMC + NAC, or BDMC. (**C** and **D**) The transwell invasion assay was performed to determine the effect of icotinib plus BDMC treatment on cell invasion. After 24 h incubation with or without the indicated concentration of icotinib and/or BDMC, or the two combinations plus NAC, H460 and H1781 cells that had invaded the lower chamber were fixed, stained, and counted using light microscope or fluorescent microscopy-based high content screening system, as described in Materials and Methods. The quantity of invasion cells was defined as 100% in control. *P < 0.05 versus icotinib + BDMC + NAC, or BDMC. (**E**) H460 and H1760 cells were treated with icotinib and/or BDMC, or the two combinations plus NAC at indicated doses, and then subjected to immunoblot assay to determine the protein expressions as indicated. (**F**) After transfection with siControl, or siVDAC1, or siBeclin-1, H460 and H1781 cells were treated with icotinib plus BDMC at indicated doses for 48 h, and then subjected to immunblot assay to determine the protein expressions as indicated.

Since curcumin can inhibit lung cancer cell migration and invasion 28,29, we decided to investigate the effect of icotinib plus BDMC treatment on migration and invasion abilities of H460 and H1781 cells by the wound-healing assay and transwell assay. As compared with icotinib or BDMC alone, the two combination significantly suppressed migrations of the two NSCLC cell lines (Figure 6A and 6B). Likewise, icotinib plus BDMC treatment markedly repressed the cell invasion ability in a synergistic manner compared to single-drug treatment (Figure 6C and 6D). These effects were abated by NAC addition, implying that the two drug combination-caused reductions of cell migration and invasion abilities partially depend on ROS generation. Curcumin was reported to inhibit cancer cell migration and invasion through activation of the tumor suppressor DanJ-like heat shock protein 40 (HLJ1) which is modulated by JunD, one of activator protein (AP-1) components 29. HLJ1 be also able to regulate cell migration and invasion via E-cadherin 30. Thus we evaluated the effect of BDMC plus icotinib on these protein expressions. We found that the two drug combination clearly upregulated expressions of E-cadherin, JunD and HLJ1 and downregulated expressions of MMP-2, MMP-9 and VEGF, which were attanuated by adding NAC (Figure 6E). Moreover, knockdown of VDAC1 or Becine-1 retarded the combination treatment induced alterations of these proteins expressions (Figure 6F). Taken together, the findings indicate that reductions of cell migration and invasion abilities caused by icotinib plus BDMC is mediated by repressing MMP-2, MMP-9 and VEGF and upregulating of E-cadherin, JunD and HLJ1, this process is associated with oxidative stress activation and autophagy induction in the TKI-resistant NSCLC cells.

### Icotinib and BDMC combination surmount icotinib resistance *in vivo* through dual induction of autophagy and apoptosis

**Figure 7:**
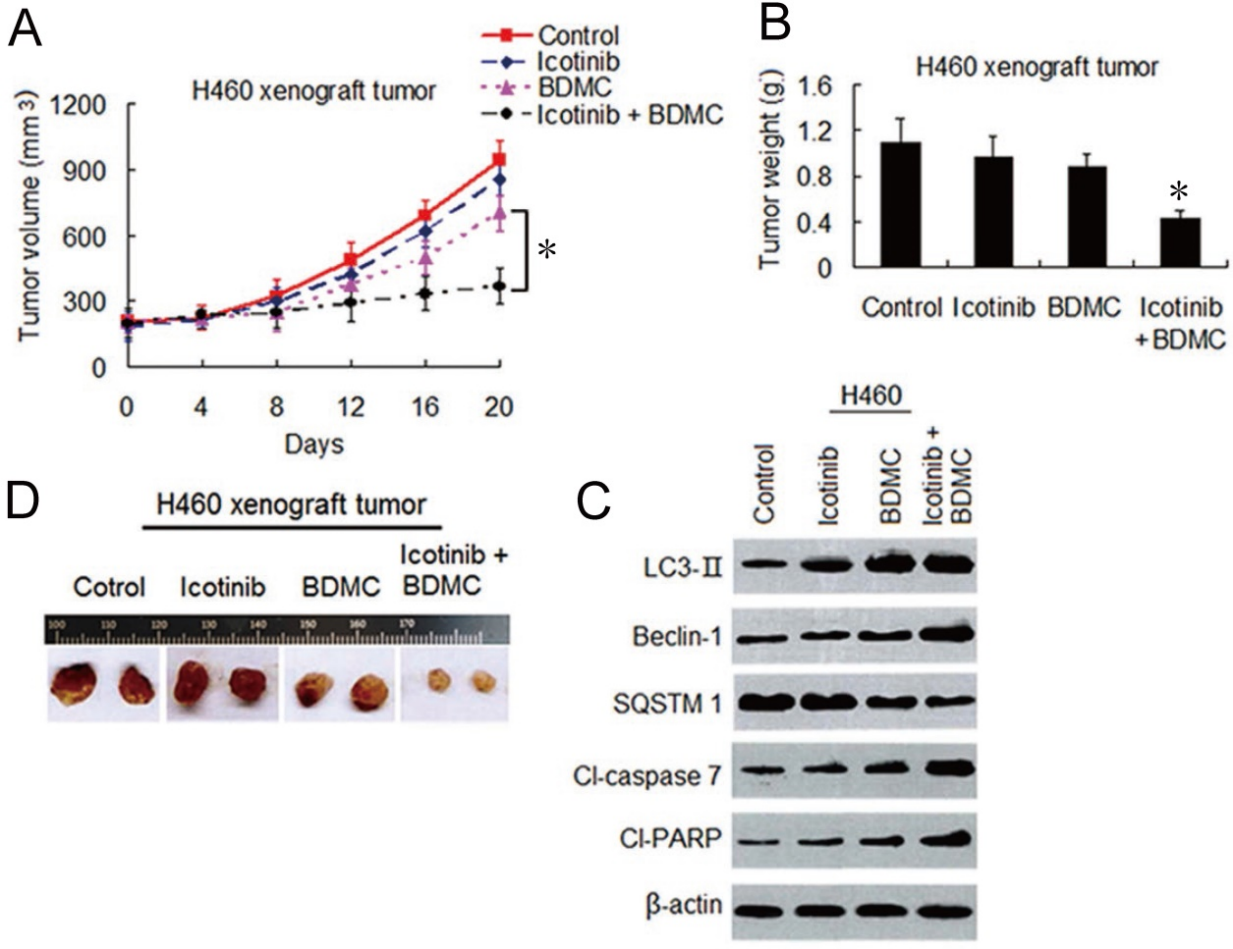
** BDMC enhances anti-tumor effect of icotinib *in vivo* through dual induction of autophagy and apoptosis. (A** and** B)** After H460 cell mouse xenograft models were established, the mice were treated with vehicle (control), icotinib, or BDMC, or the two drug combination respectively. Tumor volume was measured at the time indicated after the onset of treatment. (*P < 0.05). The tumor weight was measured at the end of the experiment (*P < 0.05 vs icotinib, or BDMC treatment alone). (**C**) Representive pictures of tumor samples from the mice bearing H460 cell tumors receiving different treatments as indicated. (**D**) Immunoblot analysis of the expressions of proteins as indicated in H460 cell tumor samples from the mice in each treatment group. All experiments were repeated three times.

To evaluate the effects of this combination strategy *in vivo*, we established H460 cell xenograft mouse models as described in materials and methods. As shown in Figure 7A-7C, icotinib alone had hardly effect on growth of the tumors. BDMC alone had only minor influence on growth inhibition of the tumor. In contrary, combination treatment with icotinib and BDMC significantly inhibited tumor growth compared with either of the two drugs. Immunoblot assay showed that combination treatment clearly upregulated expressions of LC3-, Beclin-1, cleavad-caspase 7 and -PARP, and downregulated SQSTM1 expression compared to single treatment in xenograft tumor specimens (Figure 7D), which were in consistent with the finding *in vitro* (Figure 4C and 4F). These results further demonstrated that BDMC can sensitize NSCLC cells to icotinib, and thereby surmounting icotinib resistance in NSCLC cells through dual induction of autophagy and apoptosis.

## Discussion

In this study, we identified BDMC as a sensitizer of icotinib by enhancing lethal effect of icotinib on EGFR-TKI-resistant NSCLC cells. Although low doses of icotinib and BDMC alone slightly inhibited the survival of these NSCLC cells, combination of the two drugs led to more significant reduction of cell viability than single drug treatment. CI data analysis indicated that this combination is highly synergistic in suppressing cell growth. Parallel to this, icotinib in combination with BDMC caused marked decrease of EGFR activity through depressing Sp1 and the interaction of Sp1 and HDAC1/HDCA2, consequently diminishing protein and mRNA expressions of HDAC1/HDCA2 which are the key regulators for cancer development and growth 31. The decrease of EGFR activity was paralleled by reductions of EGFR downstream proteins AKT and JNK phosphorylation levels and Sp-dependent proteins Her2, c-Myc and survivin expressions, which are in line with the notion that the hyperactivated AKT pathway and increased expressions of Her2, c-Myc and survivin are associated with resistance to EGFR-TKI in NSCLC 32,33. Also, we showed that icotinib plus BDMC treatment caused growth inhibitory effect in the NSCLC cells was accompanied by a synergistic increase of intracellular ROS levels. Notably, co-treatment with the free radical scavenger NAC diminished the lethality of the NSCLC cells by the two drugs, suggesting a causal role for oxidative stress in facilitating cell death. Furthermore, we found that the upregulation of VDCA1 expression induced by icotinib plus BDMC could be functionally involved in oxidative stress dependent-apoptosis. VDAC1 knockdown has been found to be able to suppress ROS generation and consequently, limits cytochrome c release to the cytoplasm 34. Indeed, we effectively diminished the increase in ROS levels induced by the two drug combination in the cells where VDAC1 was knocked down. Simultaneously, the increase of apoptosis cells by the combination treatment was also reversed partially by silencing VDAC1. VDAC1 pro-apoptotic effects are also regulated by interactions with anti-apoptosis proteins such as the enzyme hexokinase (HK), or Bcl-2. Especially, the binding of VDAC1 to HK1 provides a metabolic benefit to cancer cells and inhibits pro-apoptotic capacity by blocking ROS production and cytochrome c release 23. Some studies showed an opposite effect of VDAC2 which bind to the Bak protein and preventing Bak-dependent mitochondrial apoptosis 35. We also found the decreases of HK1 and VDAC2 expressions in the TKI-resistant NSCLC cells treated with icofinib plus BDMC compared with untreated or single drug treated cells, revealing the convergent mechanisms that potentiate mitochondria-dependent apoptosis by targeting VDAC proteins.

Although icotinib alone caused autophagy induction to a small extent in TKI-resistant cells, combining with BDMC obviously raised autophagy levels. Importantly, reduced cell viability by icotinib plus BDMC treatment can be reversed partially by CQ or 3-MA, two autophagy inhibitor, indicating that the reduction of cell viability was dependent on autophagy. Furthermore, increased autophagy induction by the combination treatment was retarded by NAC and enhanced by MIT, suggesting that the autophagy induction is associated with ROS generation and inhibition of Sp1 and HDAC1/HDCA2 interaction. A growing evidence indicate that downregulation of Sp1 and Sp3 expressions by drugs or RNA interference can induce autophagy by depressing EGFR activity and downstream the AKT/MAPK pathways in cancer cells 20,36. Also HDAC inhibition promote autophagy induction through multiple mechanisms including activation of oxidative stress 37, and oxidative stress is considered as activating force for autophagy induction 25. Thus, the combination of icotinib and BDMC might create oxidative stress-related microenvironments favorable for autophagy and elevated oxidative burden drives autophagy moving forward 38. Interestingly, apoptosis reaction by the two drugs combination was attenuated by CQ, 3-MA and NAC, or knockdown of Beclin-1, signifying that icotinib plus BDMC induced autophagy-related apoptosis and this effect is associated with oxidative stress. On the other hand, the two drug combination-induced apoptotic cell death might be not completely dependent on autophagy, as the increase of apoptotic cells induced by icotinib plus BDMC were prevented, only in part; by co-treatment with autophagy inhibitors CQ or 3-MA. Therefore, the mechanisms of cell death caused by icotinib plus BDMC might include autophagic cell death, autophagy-dependent and -independent apoptotic cell death.

BDMC was found to induce rapid DNA damage in cancer cells 12. In this study, icotinib plus BDMC induced more profound DNA damage than that produced by either of the two, which was blocked by 3-MA or NAC, indicating that the synergistic induction of DNA damage by icotinib plus BDMC is autophagy and oxidative stress dependent. It is known that there are crosstalk between autophagy and DNA damage in cancer cells upon genotoxic agent exposure 39. DNA damage can activate autophagy through various DNA damage responses signaling at multiple levels. Also, autophagy is directly involved in DNA damage repair 38,40. Considering that autophagy inhibitor 3-MA partially retarded DNA damage induced by icotinib plus BDMC, it is possible that icotinib and BDMC combination also directly induced DNA damage to a certain extent.

Curcumin was reported to inhibit NSCLC cell migration and invasion through upregulation of HLJ1 expression mediated by JNK/JunD pathway 29, which is in line with our results using BDMC. Co-treatment with NAC prevents this effect caused by icotinib plus BDMC, suggesting that ROS suppression is involved in the reductions of migration and invasion abilities in the cells. HLJ1 is considered to be a cancer suppressor and one of the selected candidate targets for control of metastasis and invasion 29. E-cadherin, as the key component for adherence junctions between epithelial cells, has been considered as a suppressor molecule of migration and invasion, and cancer cells with decreased E-cadherin expression are more likely to detach from a tumor mass, resulting in tumor metastasis 41. HLJ1 might regulate cell invasion and migration through E-cadherin 30. MM-2 and MMP-9 have been found to have an important role in basement membrane type II collagen degradation 42. The present study reveals that icotinib and BDMC combination suppress migration and invasion in TKI-resistant NSCLC cells through the induction of HLJ1/E-cadherin and the inhibition of MMP-2/MMP-9 expression, which depend on ROS generation and autophagy induction.

In conclusion, this study is the first time to describe that BDMC in combination with icotinib produced synergistic growth inhibitory effect on primary EGFR-TKI resistant NSCLC cells, through inductions of autophagic cell death, autophagy-dependent and -independent apoptosis. Therefore, the combination of icotinib and BDMC in a selected population of NSCLC patients with EGFR wild-type and K-ras mutation or Her2 mutation, who often refractory to EGFR-TKIs might represent a valid treatment option that warrants clinical investigation.

## Supplementary Material

Supplementary materials and methods, figures.
