# Supplementary material for: Bisdemethoxycurcumin Enhances the Sensitivity of Non-small Cell Lung Cancer Cells to Icotinib via Dual Induction of Autophagy and Apoptosis
Source: Int J Biol Sci. 2020 Mar 5;16(9):1536–50. doi: 10.7150/ijbs.40042 (PMC7097919; doi:10.7150/ijbs.40042)
Supplement: Supplementary file 1 — Supplementary materials and methods, figures. [file ijbsv16p1536s1.pdf]

Bisdemethoxycurcumin enhances the sensitivity of non-small cell lung cancer cells to icotinib via dual induction of autophagy and apoptosis

Min Xiang, He-Guo Jiang, Yang Shu, Yu-Jiao Chen, Jun Jin, Yu-Min Zhu, Mei-Yu Li, Jian-Nong Wu, Jian Li

Supplementary material and methods

Agents and antibodies

Icotinib was purchased from Betta Pharmaceutic Co., Ltd (Zhejiang, China). BDMC, EGF, 3-methyladenine (3-MA), N-acetylcystein (NAC), chloroquine (CQ), and mithramycin A (MIT) were purchased from Sigma-Aldrich (St. Louis, MO). Antibodies against EGFR, p-EGFR (Y1068), AKT, p-AKT (S473), JNK, p-JNK (T183/185), Sp1, Sp3, HDAC1, HDAC2, HDAC3, HDAC4, Ac-H3, H3, VDAC1, VDAC2, hexokinase1 (HK1), KEAP1, NRF2, glycogen synthase kinase 3β (GSK 3β), cytochrome C, and β-actin were from Santa Cruz Biotechnology (Santa Cruz, CA). Antibodies against Her 2, c-Met, Survivin, c-Myc, microtubule-associated protein 1 light chain 3-I (LC3-Ⅰ), LC3-Ⅱ, SQSTM1, Beclin 1, caspase-7, cleaved-caspase-7, poly (ADP-ribose) polymerase (PARP), cleaved-PARP, ATM, p-ATM (S1981), Chk1, p-Chk1 (S345), AKP, p-AKP (S824), H2AX, γ-H2AX (S139), VEGF, MMP-2, MMP-9, E-cadherin, Jun D and HLJ1 were from Abcam Inc (Combride, MA).

Primary antibodies of p-ATM (S1981) and 53BP1 and second antibodies Alexa Fluor 568 anti-mouse IgG and Alexa Fluor 568 anti-rabbit antibodies were from Jackson, ImmunoResearch (Lancaster, PA).

Cell survival and viability

For cell survival detection, cells were seeded in 96-well plates at an initial density of 2×10^3/well and incubated for 24 hours. Then the cells were treated with the indicated drugs at various doses and were incubated for another 48 hours. Subsequently, the cells were washed with PBS, fixed with 3.7% formaldehyde for 30 minutes and stained with 1.0% methylene blue for 30 minutes. Next, the plates were rinsed in running water and left to dry. 100 μl solvent (10% acetic acid, 50% methanol and 40% H_2O) was added to each well to dissolve the cells after which the optical density (OD) of the released color was read at 660 nm. The relative cell survival rates were calculated with the values of mock-treated cells set at 100%. Cell viability was determined using the cell counting kit-8 (CCK-8) assay according to manufacturer`s instructions. Briefly, cells were seed at a density of 3×10^4 cells per well in 96-well plates, and allowed to grow in the growth medium for 24 hours. Cells were then treated with indicated drugs. Two day after drugs addition, 10μl of cell proliferation reagent CCK-8 (DOJNDO, Japan) was added into media in each well and the cell were incubated for 2 hr at 37℃. The absorbance of each well was determined with a spectrophotometer reading at a wavelength of 450nm. Absorbance is assumed to be directly proportional to the number of viable cells.

Clonogenic assay and cell apoptosis analysis

For clonogenic assay, cells plated in 6-well plates were treated with various drugs at the doses indicated. After 72 h, cells were washed with PBS and trypsinized. Then cells were mixed in 1.0 ml of 0.3% McCoy's agar containing 10% FBS. The cultures were maintained in a 37°C, 5% CO2 incubator for 14 days. After being fixed with 4% paraformaldehyde for 15 min, the cells were stained with 0.1% crystal violet for 15 min before washing with tap water and air-drying. The colony formation rate was calculated with the following formula: colony formation (%) = (number of colonies treated with drugs/number of control colonies) ×100%.

Cell sub-G1 population was analyzed by flow cytometry. Briefly, cells were treated with the indicated drugs for 48 h, harvested and fixed using 7% ethanol. And then cells were stained with mixture of 50 ug/mL propidium iodide, 0.2 % Triton X-100, and 100 ug/mL RNAase, and performed using a FACS flow cytometer equipped with Modifit LT for Mac V 2.0 software (BD BioScience, NJ).

Immunoblot assay and real-time quantitative PCR analysis

For immunoblot assay, total cell extracts were obtained by direct lysis of cells in lysis buffer (50 mM Tris HCl, 150mM NaCl, 10mM β-glycerophosphate, 1% NP-40, 1μg/ml aprotinine, 1μg/ml leupeptine, 1μg/ml pepstatine, 0.5mg/ml pefabloc, and 1mM Na3VO4) boiled for 5min, and subjected to polyacrylamide SDS gel electrophoresis (Sigma Co., USA). After electrophoresis, proteins were transfer to Immobilonp-FL transfer membranes (Sigma Co., USA). The membranes were then

blocked with 5% nonfat dried milk in PBS-T overnight at 4℃ and then incubated at room temperature for 1 hr or overnight at 4℃in appropriate primary antibody diluted in PBS-T. After three times 5 min washes in PBS-T, membranes were then incubated for 1 hr at room temperature in appropriate secondary antibody at a dilution of 1:2000 in PBS-T + 0.5% milk. Finally, blots were washed five times for 5 min in PBS-T, and films were developed by using the ECL Western Blot Analysis System (Beijing Sage Creation Technology Co., Ltd, China).

For quantitative PCR analysis, total RNA was extracted from cell samples using Trizol reagents (Invitrogen) following the manufacture's instructions. RNA was reverse transcribed into cDNA using qScript (Quanta Biosciences) following the manufacture's instructions and the reactions were run on an ABI 7500 Fast real-time PCR systems. Detection of PCR products was accomplished by measuring the emitting fluorescence (Rn) at the end of each reaction step. Threshold cycle (Ct) corresponds with the cycle number required to detect a fluorescence signal above the baseline. mRNA relative quantification was calculated with the Ct ($2^{-\Delta\Delta Ct}$) method. In accordance with the method, the mRNA amounts of a target gene were normalized to an endogenous control and relative to a calibrator. $\Delta\Delta C_T$ represent the difference between the mean ΔC_T value of the cells tested and the mean ΔC_T value of the calibrator, both calculated after the same PCR run, whereas ΔC_T is the difference between the C_T of the target gene and the C_T of the endogenous reference gene (β-actin) of the same sample. The relative quantitative value was expressed as $2^{-\Delta\Delta Ct}$.

MDC staining and immunofluorescence

The formation of acidic vesicular organelles (AVOs) was detected using monodansylcadaverine (MDC) staining. Briefly, cells were seeded on the 24-well plates and treated various drug at the indicated doses for 48 h. Following treatment, the cells were incubated with 0.5 mM MDC for 2 h in serum-free medium at room temperature, then the cellular fluorescent changes were measured at an excitation of 380 nm and an emission of 510 nm and observed under an epi-fluorescence microscope (Olympus, Tokyo, Japan). For immunofluorescence analysis of γ-H2AX, p-ATM and 53BP1 foci, cells were treated with the indicated drugs for given time, and fixed by 4% paraformaldehyde for 15 min, followed by permeabilization with 0.1% NP-40 in PBS for 15 min. Then cells were stained with primary mouse anti-human γH2AX, or p-ATM, or 53BP1 antibodies and second antibodies Alexa Fluor 568 anti-mouse IgG and Alexa Fluor 568 anti-rabbit antibodies. Images were taken with an Ax-70 microscope (Olympus) and analyzed using Image-Pro software (Medica Cybernetics). Each experiment was performed in triplicate.

Cell migration and invasion assays

Cell migration was determined by wound healing assay, cells were cultured in 24-well plates and grown in medium containing 10% FBS to nearly confluent cell monolayer, then carefully scratched using pipette tips to create a denuded zone (gap) of constant width. Subsequently, cellular debris were washed with PBS, and the cells were exposed to various drugs at the doses as indicated. The wound closure was monitored

and photographed at 0 and 36 h under a Levia inverted microsccope (Olympus, Tokyo, Japan). To quantify the migrated cells (the percentage of migrated cells in control is designed as 100%), pictures of the initial wounded monolayers were compared with the corresponding picture of cells at the end of the incubation .

Cell invasion was examined by membrane transwell culture system.Ttranswll membrane (8-µm pore size, 6.5-mm diameter; Corning Castar Corporation, MA), coated with Matrigel (BD BioScience Discovery Labware) was used for invasion assay. Cells were trypsinized, centrifuged, and resuspended at 10^7 cells/mL in DMEM. Cells were seeded on the upper wells of precoated transwell, 2×10^4 cells per well. Lower wells of the transwells contained the same medium with 10% FBS. After 48 h of incubation with various drugs at doses as indicated, the cells on the upper well and the membrances coated with Mafrigel were swabbed with a Q-tip, fixed with methanol, and stained with 20 % Giemsa solution. The cells that were attached to the lower surface of the polycarbonate filter were counted under an inverted microscope (the percentage of invaded cells in control is designed as 100%).

Supplementary Figures

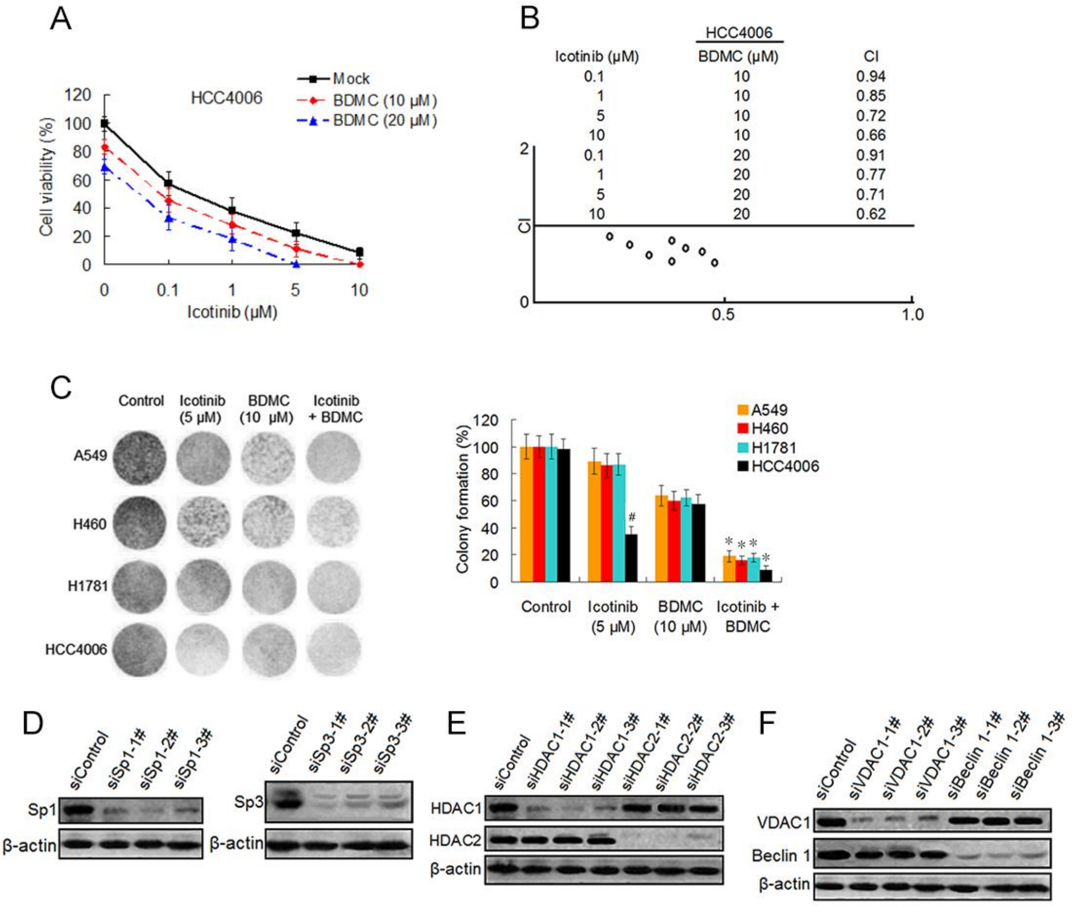

Figure S1 BDMC limitedly enhances icotinib sensitivity in EGFR-TKI-sensitive NSCLC cell line HCC4006. (**A** and **B**) HCC4006 cells were treated with icotinib or BDMC alone, or the two combination at indicated doses for 48 h . Then cell viability was detected by CCK-8 assay. The results was determined as the percentage surviving cells in drug-treated cultures relative to DMSO-treated control cells. CI values for the combination of icotinib and BDMC were calculated using the Calcusyn software (Cambrige, UK) as described in Materials and Methods. (**C**) A549, H460, H1791 and H4006 cell lines were treated with indicated doses of single or drug combination for

12 days. Colonies were stained with 0.1% crystal violet and photographed. The percentage of clonogenic survival was calculated by comparing the control (designated as 100%). **P* < 0.05 vs BDMC and icotinib; #*P* < 0.05 vs A549， H460 and H1781. (**D**) Representative images of the protein expressions after silencing of Sp1, Sp3, HDAC1, HDAC2, VDAC1 and Beclin-1 genes. Following transfection with siRNAs against Sp1, Sp3, HDAC1, HDAC2, VDAC1 or Beclin-1, whole cell lysate was prepared from H460 cells and subjected to immunoblot assay to determine the expressions of corresponding protein expressions.

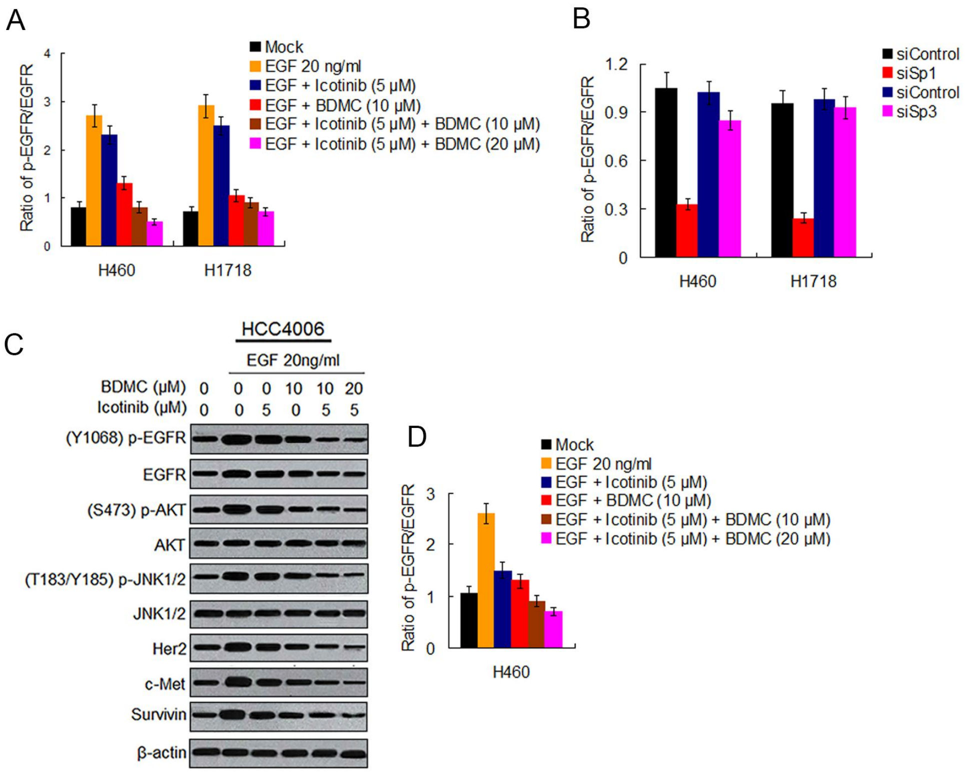

Figure S2 (**A**) Quantification analysis of phosphorylated (p)-EGFR/EGFR ratio in H460 and H1718 cells from immumoblot assays after treatment with indicated drugs presented in Figure 2A. The ratio of p-EGFR/EGFR was determined by a densitometric analysis. (**B**) The ratio of p-EGFR/EGFR in H460 and H1718 cells from immumoblot assays after treatment with indicated drugs presented in Figure 2B. (**C** and **D**) HCC4006 cells were pre-treated with icotinib or BDMC, or the two combination at indicated doses for 6 h, and then stimulated with EGF at indicated doses for 1h. Then cell lysates were prepared and subjected to immunoblot assay to determine the protein expression as indicated. Ratio of p-EGFR/EGFR was analyzed using a densitometry.

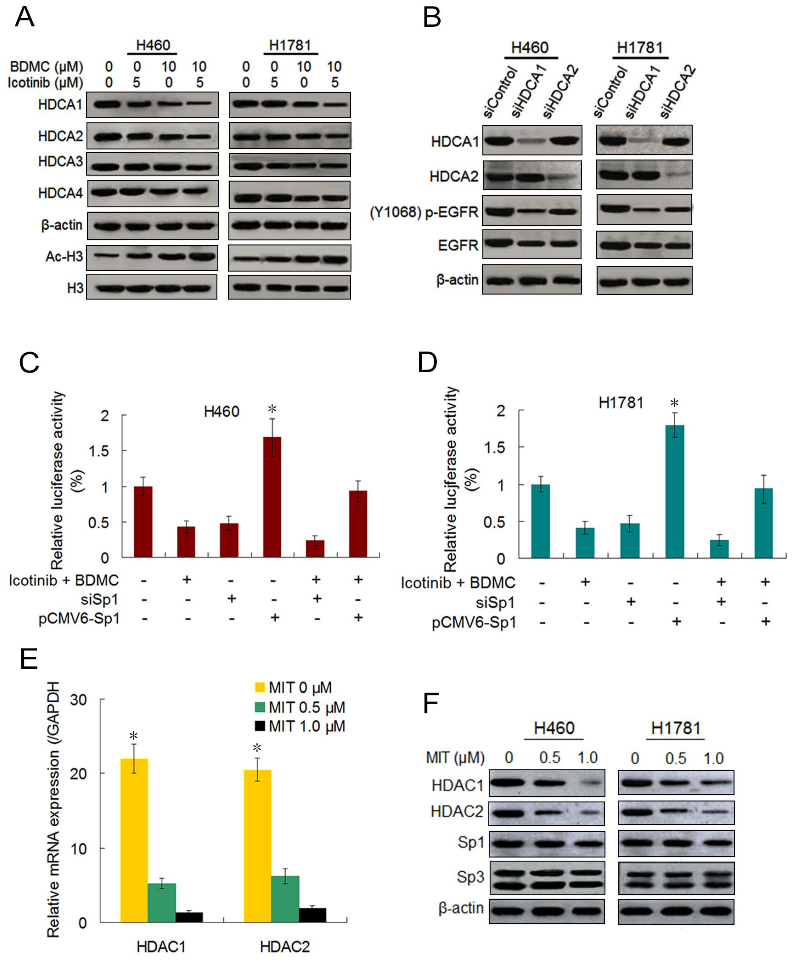

Figure S3 Combination of icotinib and BDMC suppresses EGFR activity by downregulating expressions of Sp1 and HDAC1/HDAC2. (**A**) H460 and H1781 cells were treated with indicated doses of single drugs or drug combination for 48 h, then subjected to immunoblot analysis to detect protein expressions as indicated. (**B**) Expressions of HDCA1 and other indicated proteins in control and HDCA1 and HDCA2 knocked down cells were determined by immunoblot analysis in H460 and H1781 cells. (**C** and **D**) H460 and H1781 cells were treated with icotinib (5 μM) plus BDMC (10 μM), or transfected with siSp1 or plasmid containing Sp1 (PCMV6-SP1), or treated with icotinib plus BDMC in combination with siSp1 transfection, or with

Sp1 plasmid (PCMV6-Sp1) transfection, and HDCA1 promotor activity was determined by luciferase assay as described in Materials and Methods. *$P < 0.05$ vs other every treatment. (**E**) H460 cells were treated with MIT at indicated doses for 48 h. Then HDAC1 and HDAC2 mRNAs were measured by real-time quantitative PCR. *$P < 0.05$ vs MIT 0.5 μM and 1.0 μM. (**F**) H460 and H1781 cells were treated with MIT at the indicated doses for 48 h, then the cell lysates were prepared and subjected to immunoblot assay to detect protein expressions as indicated.

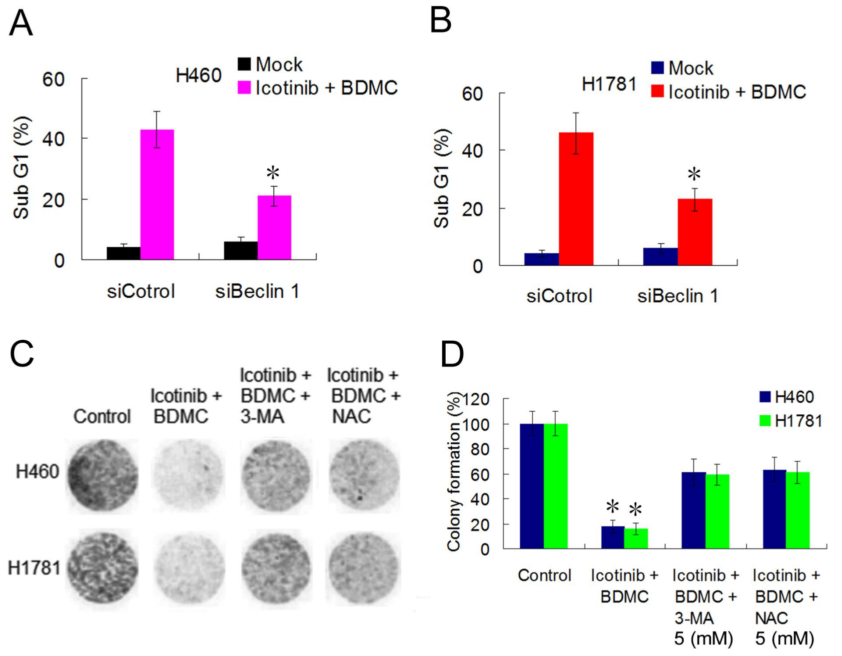

Figure S4 Combination of icotinib and BDMC induces autophagy-dependent and -undependent apoptosis in EGFR-TKI-resistany cell line. (**A** and **B**) After transfection with siControl or siBeclin-1, H460 and H1781 cells were treated with icotinib (5 μM) plus BDMC (10 μM) for 48 h, then the percentage of sub G1 cells was determined by flow cytometry. *P < 0.05 vs icotinib + BDMC in siContrl. (**C** and **D**) H460 and H1781 cells were treated with icotinib (5 μM) plus BDMC (10 μM), or the two combination plus 3-MA (5 μM), or plus NAC (5 mM) for 12 days. Colonies were stained with 0.1% crystal violet and photographed. The percentage of clonogenic survival was calculated by comparing the control (designated as 100%). *P < 0.05 vs icotinib +BDMC + 3-MA, or NAC.

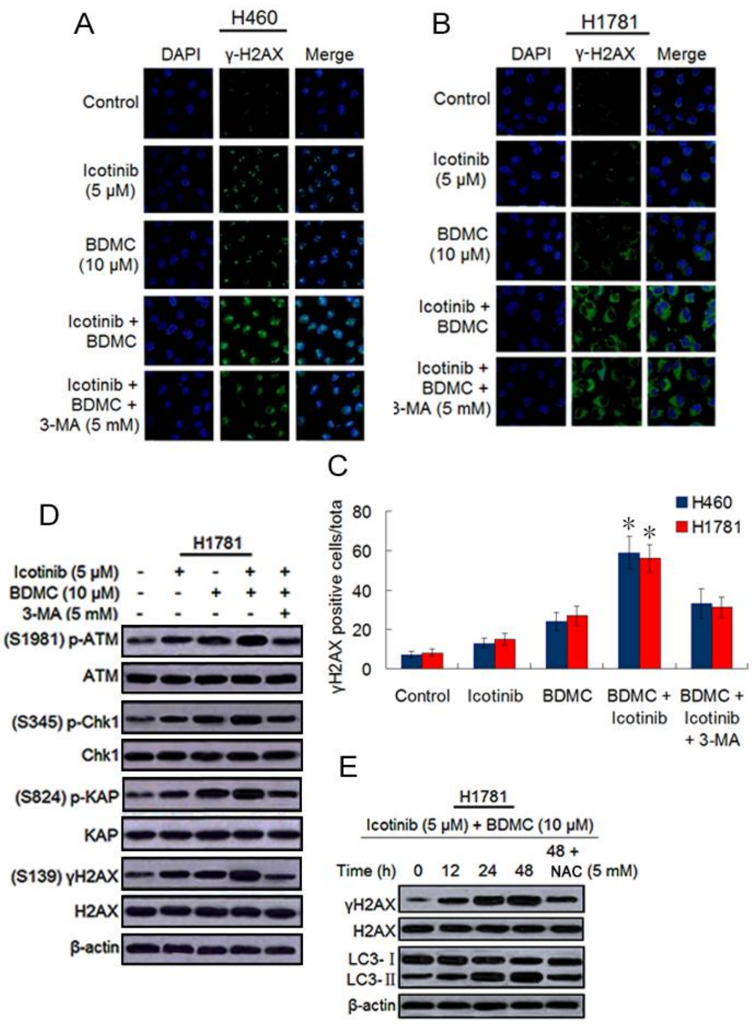

Figure S5 Icotinib and BDMC combination augments DNA damage that depend on autophagy and ROS generation. (**A, B** and **C**) H460 and H1781 cells were treated with icotinib or BDMC alone, or their combination, or the combination plus 3-MA at indicated doses for 48 h. Then cells were fixed and immunostained with an anti-γ-H2AX antibody. The percentage of γ-H2AX foci positive cells were quantified using Matafer Software. *P < 0.05 vs icotinib + BDMC + 3-MA, or BDMC. (**D**) H1781 cells were treated with icotinib or BDMC, or their combination, or the combination plus 3-MA at indicated doses for 48 h. Then cell lysates were prepared and subjected to immunoblot analysis to detect phosphorylated (p) and total protein

expressions as indicated. (**E**) H1781 cells were treated with icotinib plus BDMC at the indicated doses for 12, 24 and 48 h, or the combination plus NAC for 48 h. Then immunoblot assay was performed to determine protein expressions as indicated.
